# Taiwan Government-Guided Strategies Contributed to Combating and Controlling COVID-19 Pandemic

**DOI:** 10.3389/fpubh.2020.547423

**Published:** 2020-10-21

**Authors:** Chung-Chu Chen, Cheng-Yin Tseng, Wai-Mau Choi, Ya-Chun Lee, Tsung-Hsien Su, Chin-Yi Hsieh, Chih-Ming Chang, Shun-Long Weng, Po-Huang Liu, Yu-Lin Tai, Chien-Yu Lin

**Affiliations:** ^1^Department of Internal Medicine, Hsinchu MacKay Memorial Hospital, Hsinchu, Taiwan; ^2^Teaching Center of Natural Science, Minghsin University of Science and Technology, Hsinchu, Taiwan; ^3^Center of Infection Control, Hsinchu MacKay Memorial Hospital, Hsinchu, Taiwan; ^4^Department of Emergency Medicine, Hsinchu MacKay Memorial Hospital, Hsinchu, Taiwan; ^5^Department of Obstetrics and Gynecology, Hsinchu MacKay Memorial Hospital, Hsinchu, Taiwan; ^6^Department of Medicine, MacKay Medical College, New Taipei, Taiwan; ^7^Department of Nursing, Hsinchu MacKay Memorial Hospital, Hsinchu, Taiwan; ^8^Department of Pediatrics, Hsinchu MacKay Memorial Hospital, Hsinchu, Taiwan

**Keywords:** novel coronavirus, COVID-19, SARS-CoV-2, quarantine, 2019-nCoV, pandemic, government strategy

## Abstract

Coronavirus disease 2019 (COVID-19) is highly contagious, and thus has become an emerging health crisis worldwide. The optimal strategies to prevent the spread of this disease are inconclusive, and therefore, the adopted measurements to combat COVID-19 varies in different countries. In mid-March and late-August 2020, we performed internet searches to collect relevant information, from sources such as the website of the World Health Organization. The epidemiological data of COVID-19 from several countries were collected and we found that Taiwan had a comparably successful story for combating the pandemic. As of mid-March, Taiwan had high rates of diagnostic testing (688.5 tests per million citizens) with a lower infection rate (49 cases, 2.1 cases per million people). As of late-August, there were 488 cases (20 cases per million people). Furthermore, Taiwanese government-guided strategies and hospital data were also reviewed. We summarized some important strategies to combat COVID-19, which include: (1) border control; (2) official media channel and press conferences; (3) name-based rationing system for medical masks; (4) TOCC-based rapid triage, outdoor clinics, and protective sampling devices; and (5) social distancing, delaying the start of new semesters, and religious assembly restriction. In conclusion, Taiwan had lower rates of COVID-19 compared with other countries, and Taiwan government-guided strategies contributed to the control of the disease's spread.

## Introduction

In December 2019, novel coronavirus disease (COVID-19), caused by Severe Acute Respiratory Syndrome Coronavirus 2 (SARS-CoV-2), was detected in central China and then spread throughout the country and to the rest of the world rapidly (1–4). The number of infected people increased exponentially, and more than 150,000 confirmed cases were reported by mid-March 2020; the phenomenon was officially recognized as a pandemic by the World Health Organization (WHO) on 11 March 2020 (1, 5). The basic reproductive value of the virus was estimated at 2-3, and the case fatality rate was ~3% (1, 6–8). There are no effective antiviral drugs or vaccines available. The clinical manifestations are protean, and it is estimated that 80% of infected people are asymptomatic or experience mild cases; therefore, it is difficult to detect potentially infected patients at an early stage and disrupt the transmission chains (9–11). International cooperation is especially crucial to combat this disease.

Due to the recent emergence of SARS-CoV-2 in humans, researchers are developing best practices in real-time to combat this new virus. Several strategies were adopted very early to block transmission, including the unprecedented lockdown of Hubei and other provinces, and travel bans within China and many other countries globally (1, 6, 12). But geographic characteristics, medical resources, population densities, and social norms vary widely across countries and the adopted strategies facing COVID-19 differ widely too. Based on the observed high degree of contagiousness of SARS-CoV-2, blocking the spread of this disease poses steep challenges. However, by focusing upon slowing down the speed of transmission and flattening the curve of coronavirus cases, governments have been able to prevent the collapse of their medical systems, reduce mortality, and buy time for the development of antiviral drugs and vaccinations (13). Furthermore, technological advances may be beneficial in combating the pandemic. Big data analytics, new technology, and proactive testing have been applied in Taiwan to combat COVID-19 (14, 15). These strategies may be collectively beneficial in reducing virus transmission.

Taiwan is a small and populous island which is geographically very close to China. Social interaction between China and Taiwan is frequent, thus leading to a high risk of virus transmission. Central Epidemic Command Center (CECC) was assembled to combat the COVID-19 pandemic in Taiwan on 20 January 2020. The commander of the CECC was the Taiwanese Ministry of Health and Welfare minister, and the organization's members included experts from various fields. The CECC executed several strategies to reduce disease transmission and its government-guided strategies may have contributed to mitigating the disease's spread. Taiwan's first confirmed case was detected on 21 January 2020, and by mid-March there were 49 cases (16). As of late August, there were more than 25 million confirmed cases worldwide; but Taiwan has a lower incidence of infection with only 488 cases reported in total. We conducted this retrospective study to investigate the incidence of COVID-19 in some countries and the varying effects of government-guided strategies. Reviewing these measurements and experiences may be helpful for policy makers and healthcare providers.

## Study Design and Data Collection

### Study Design and Incidences of COVID-19

Our study was approved by the ethical committee of MacKay Memorial Hospital, Taipei, Taiwan (registry number: 20MMHIS140e). As of mid-March 2020, we prospectively searched the websites of WHO, Taiwan Centers for Disease Control (CDC)—which is a key department under the Ministry of Health and Welfare and is responsible for disease prevention and control—and other websites to extract data regarding patient numbers and diagnostic tests of COVID-19 in some countries based on the epidemic conditions and completeness of publicly available data (1, 16–18). The total population of each country was also obtained to calculate the case numbers per million citizens. The trend chart was also plotted.

### Government-Guided Strategies and Hospital-Adopted Measurements

Taiwan's CECC delivered information to the populace via public broadcasting; this included television, newspaper, and the internet (16). Additionally, official documents were provided to each hospital or healthcare clinic. In accordance with the government-guided strategies, individual healthcare units adapted the optimal measurements according to their own equipment, patient characteristics, human resources, and clinical settings. We retrospectively reviewed the government-guided strategies and our hospital measurements. Some photos are provided to demonstrate practical situations.

## Results of Epidemiological Data

### Incidences of COVID-19 and Diagnostic Tests Performed

**Figure 1:**
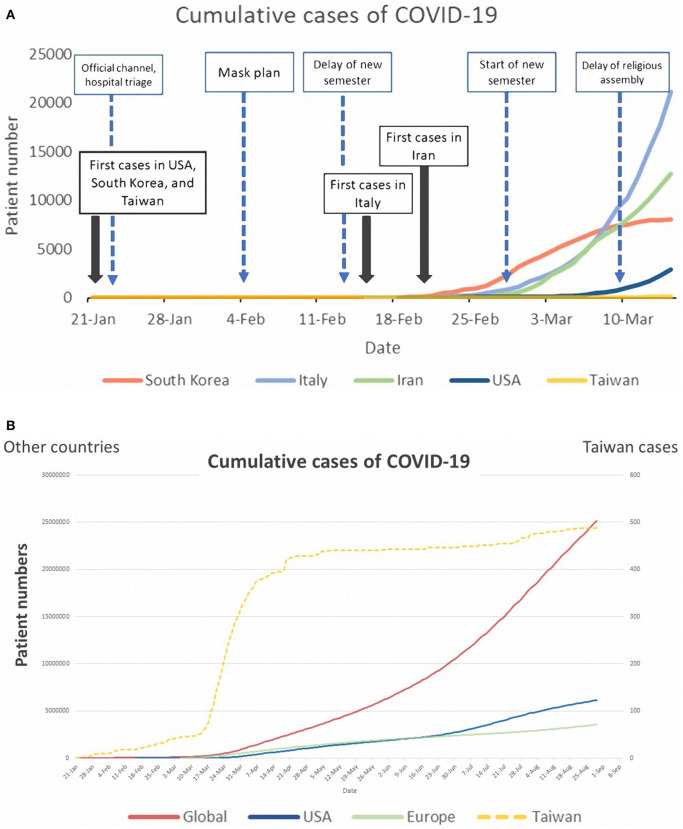
**(A)** Trend chart of cumulative cases of COVID-19 in some countries and government-guided strategies in Taiwan (as of 11 March 2020). **(B)** Trend chart of cumulative cases of COVID-19 in some countries (as of late August 2020).

As of 13 March 2020, 49 cases were confirmed as COVID-19 in Taiwan with an overall low incidence rate (2.1 cases per million citizens, Table 1). Compared with other countries, aggressive tests for COVID-19 were also observed in Taiwan and 16,089 people had received diagnostic testing (688.5 tests per million citizens). The positivity rate was relatively low (49/16 089, 0.3%). Additionally, Taiwan kept a relatively low rate of increase for disease transmission (Figure 1A). The number of infected people increased exponentially and there were more than 25 million infected people worldwide as of late August 2020 (Figure 1B). There were 488 cases in Taiwan, and Taiwan retained a low positive rate (20 cases per million citizens, Table 1).

**Table 1:** Tests for COVID-19 and positive patients in selected countries.

	**Tests performed**	**Population (millions)**	**Tests per million citizens**	**Positive cases**	**Positive test rate (%)**	**Positive cases per million citizens**
**As of 11 March 2020**						
South Korea	210,144	51.3	4,099	7,755	3.69	151.3
Italy	60,761	60.5	1,005	10,149	16.70	167.9
UK	26,261	67.9	387	456	1.73	6.7
USA	8,554	331	26	696	8.08	2.1
Japan	9,600	126.5	76	568	5.92	4.5
Taiwan	16,089	23.4	688.5	49	0.31	2.1
**As of late August 2020**						
South Korea	1,909,329	51.3	37,236	19,400	1.02	378
Italy	8,410,510	60.4	139,138	265,409	3.16	4,391
UK	16,273,209	67.9	239,511	331,644	2.04	4,881
USA	80,305,101	331	242,383	6,097,763	7.59	18,405
Japan	1,420,589	126.4	11,238	65,573	4.62	519
Taiwan	86,983	23.4	3,651	488	0.55	20

## Major Measures of Government-Guided Strategies and Hospital-Adopted Measurements

Taiwan's CECC has executed many strategies to combat COVID-19. We have summarized five important strategies and hospital-adopted measurements.

### Border Control

Many Taiwanese people either work or have business in China, and it is impossible to cut off all transportation between the two regions. With gradually decreased flights, the number of incoming people from China declined from more than 100,000 per month in 2019 to 5,000 in February 2020. At the same time, the CECC cooperated with other government departments to perform immediate border inspections. For people traveling from endemic areas, home quarantine for 14 days was required and enforced by chiefs of villages (16). If people went out without permission, they were searched by police and fined up to 300,000 New Taiwan Dollars (roughly 10,000 USD). The definition of endemic areas changed over time according to the risk assessment of the CDC.

### Official Media Channel and Press Conference

Whenever there was a new confirmed case or important policy, the CECC gave a press conference to announce the current information. As the pandemic's events changed rapidly, the frequency of press conferences was almost daily. Fear comes from ignorance and misinformation. Fake news is not uncommon in the internet era, especially during a pandemic (19, 20). Fake news causes panic, violence, unnecessary stockpiles, discrimination, and an increase in the spread of disease (21). Therefore, in Taiwan, people who release fake news are fined. An authorized and timely official channel was helpful to reduce fake news and public anxiety. Line® is one of the most popular instant messaging applications in Taiwan, thus, the CECC has built an official Line® channel to deliver correct information (Supplementary Figure 1). The application of new technology is beneficial in providing timely and correct information (14, 15).

### Name-Based Rationing System of Mask Plan

The CECC recruited all mask factories and governed the allocation of masks. For the general public, a “name-based rationing system of mask plan” was performed. Everyone could buy two masks per week at corner pharmacies. As the production capacity increased, everyone could purchase nine masks over a 2-week period beginning in April. In April, masks were donated to other countries and, starting in June, masks could be sent to people abroad. New technology was applied to the rationing plan and the purchase process was certificated by each citizen's national health insurance card. A real-time “mask map” website was also created to provide a clear instruction of storage and people could also make mask reservations using a mobile app (16) (Supplementary Figure 2).

### TOCC-Based Rapid Triage, Outdoor Clinics, and Protective Sampling Device

**Figure 2:**
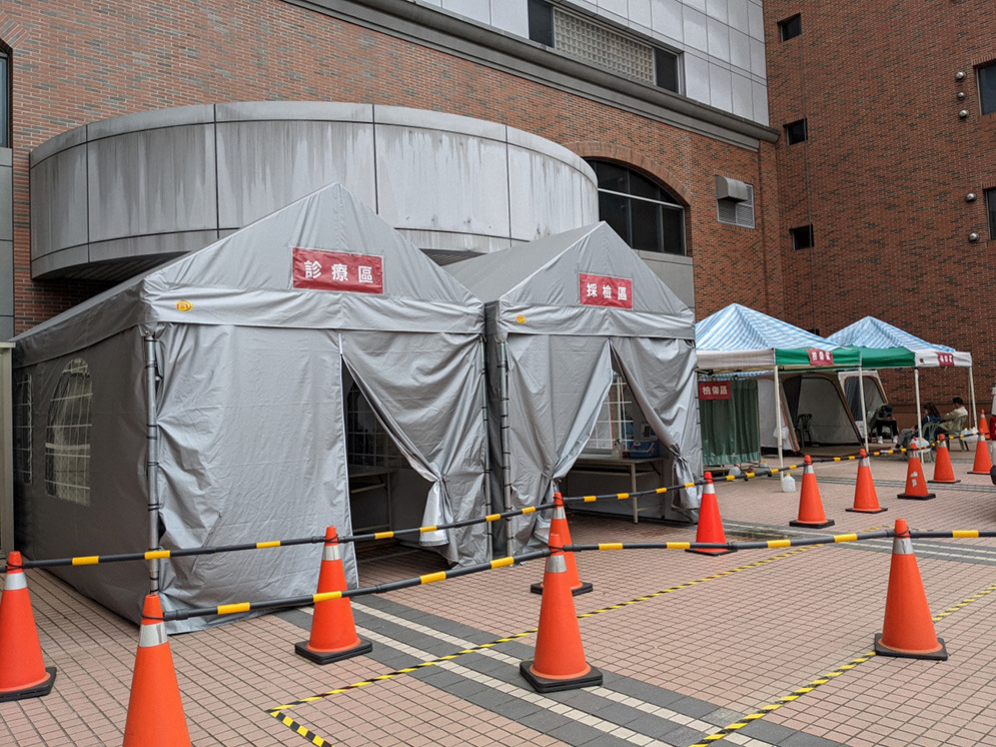
Tents as “outdoor clinics”.

**Figure 3:**
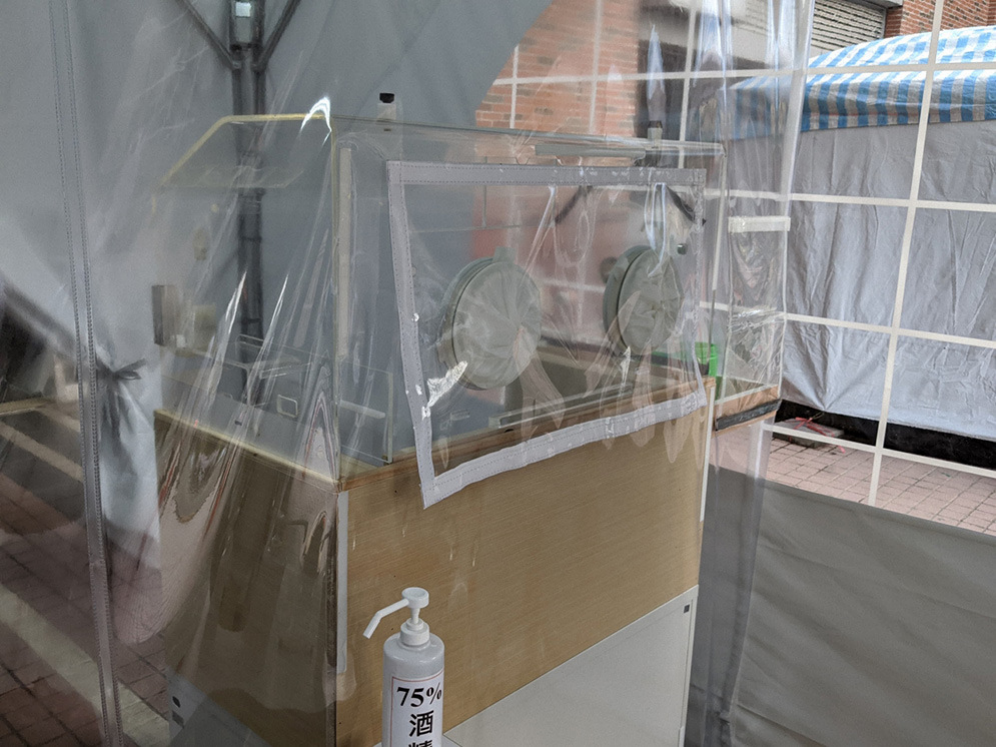
Homemade “sampling shield” for nasal swab testing.

TOCC refers to travel, occupation, contact, and cluster, which has been promoted in Taiwan for years. Patients who look for medical aid will always be asked their TOCC history. Hospitals can potentially be places for disease to spread, hence, outdoor tent clinics were set up to provide medical care for patients with travel or contact history (Figure 2). Patients with travel or contact history of COVID-19 were guided to specific outdoor clinics to prevent the virus' spread within hospitals. Inside the special outdoor clinics, healthcare providers wore medical masks, gloves, and isolation gowns to protect themselves. If COVID-19 was suspected, a nasopharyngeal swab was performed using a homemade sampling shield—or a protective cover transformed from an old incubator to offer the clinician protection against exposure (Figure 3) (22). Physicians and patients were also separated by polyvinyl chloride clear sheets. This creates a physical barrier to block the aerosols, droplets, or vomitus during sampling. Telemedicine was also used to assist in medical care and to reduce exposure. For people under quarantine having mild medical illness, such as itching skin rashes or superficial burn injuries, a history-taking and inspection was arranged via telemedicine (Supplementary Figure 3).

### Social Distancing, Delay of New Semesters, Restriction of Religious Assembly

Education and religion are essential, but crowds of any kind pose a high risk for virus transmission. In order to reduce transmission of COVID-19 in schools, the CECC cooperated with the Ministry of Education to postpone the second semester of all schools to late February. Moreover, religion is an important cultural component, but several outbreaks of COVID-19 tied to religious assembly have been reported (1). The Dajia Matsu pilgrimage is an important religious assembly in Taiwan which is listed in the UNESCO Intangible Cultural Heritage. It is usually held in late March and spans 9 days and 8 nights; millions of people participate. The religious assemblies were also postponed for months (no local cases for ~2 months) to mitigate viral transmission.

## Minor Measures, Some Details of Implementation, and Discussion

The coronavirus pandemic is a severe crisis worldwide and the optimal strategies to combat COVID-19 in each country remain unclear. Our study found Taiwan had aggressive diagnostic tests (high number of tests performed per million citizens) and low infection rates compared with other countries; in fact, it had one of the lowest incidences in the world. Government-guided strategies contributed to controlling the disease's spread and may be beneficial for reference by other countries' health policy makers and healthcare providers.

Timely quarantine and identification of infectious sources are essential to reduce virus transmission (23, 24). However, people may be afraid of quarantine and may even conceal their travel history. Taiwan's broadly covered health insurance system also shows an advantage. By rapidly connecting its medical and immigration systems, the government was able to generate a notice of travel-abroad history within 30 days to remind healthcare providers when the health insurance card was connected (16). Prompt disposition and management could be arranged. Additionally, isolation and quarantine may create huge psychological stress (25), and in order to provide humane support, a quarantine bag was delivered to people under quarantine, which included instant noodles, food, rice, facemasks, hand sanitizer, and coupons for in-home movies and music, such as Netflix and Line TV (Supplementary Figure 4). If people under quarantine felt sick, they could use a hotline (1922) to be scheduled at a designated hospital for further assistance. These measures and strategies cumulatively contributed to reduced exposure and breaking transmission chains in local transmission.

Public education with correct information about virus transmission and disease prevention is crucial during a pandemic. The CECC invited famous internet celebrities and YouTubers to make videos in various languages and to share correct information. For health personnel, teleconferences were held to share knowledge and standard procedures of medical care. All these measurements reduce unnecessary fear and panic.

The innovative “name-based rationing system for masks” was believed to have contributed to disease control during the COVID-19 pandemic (26). The major route of transmission of COVID-19 is thought to be through respiratory droplets and wearing mask is a simple and effective step to prevent infection (1, 16, 27–29). Stockpiling masks and hand sanitizer is not reasonable and is hazardous for disease control as it may cause some individuals to lack necessities (30). Taiwan's government-guided strategy to recruit mask factories and re-allocate masks ensured that healthcare providers had enough personal protective equipment (PPE) to care for highly contagious patients. PPE played a vital role in the battle against COVID-19 and shortages of PPE was an important issue. In a large-scale study in China, 3.8% of infected individuals were healthcare personnel, and the risk of healthcare-associated infection was emphasized (6). Allocation of PPE by the government and reducing stockpiling behaviors were beneficial for front-line healthcare providers. For the general public, the name-based rationing system for masks ensured everyone could have an adequate supply of masks. This strategy avoided mask stockpiling and public panic also decreased. Community transmission might be reduced when all residents wear a mask.

Unnecessary hospital visits were prohibited. Patients who look for medical aid in Taiwan are always asked for TOCC history. At the hospital entrance, a short version of COVID-19 symptoms was posted to provide a simple, graphic, and clear reminder of the disease (Supplementary Figure 5). People were asked about the purpose of their hospital visits and TOCC history was confirmed. If entry was allowed, the visitor then went through infrared thermal imagers, which decreases contact during body temperature reading (Supplementary Figure 6), and they must then apply hand sanitizer to clean their hands. These measures contributed to a decreased risk of exposure.

Our study had some limitations. First, we recognize that there is no single best strategy and that the true impact of each strategy remains unclear. The optimal strategy will differ by geographic region, culture, population density, and healthcare resources and norms. Second, due to the lack of relative quantification of the impact for each one of the components that are mentioned as part of the public health strategy, it's difficult to investigate the independent impact of each measurement.

## Conclusion

In conclusion, the emerging COVID-19 pandemic is an important health crisis worldwide. The number of infected people increased exponentially in many areas, while Taiwan experienced a relatively controllable situation. These government-guided strategies may contribute to the reduction of disease transmission.

## Data Availability Statement

All datasets generated for this study are included in the article/Supplementary Material.

## Author Contributions

C-CC, C-YT, W-MC, C-MC, and C-YL were responsible for conception. C-CC, C-YT, Y-CL, C-YH, P-HL, and Y-LT were involved in study methodology and data collection. W-MC, T-HS, and S-LW supervised study. C-YT, Y-CL, and C-MC performed data analysis. C-YL wrote the first draft. C-CC, C-YT, and W-MC contributed to this work equally. All authors approved of the manuscript.

## Conflict of Interest

The authors declare that the research was conducted in the absence of any commercial or financial relationships that could be construed as a potential conflict of interest.
