# Supplementary figures and images for: Taiwan Government-Guided Strategies Contributed to Combating and Controlling COVID-19 Pandemic
Source: Front Public Health. 2020 Oct 21;8:547423. doi: 10.3389/fpubh.2020.547423 (PMC7609768; doi:10.3389/fpubh.2020.547423)

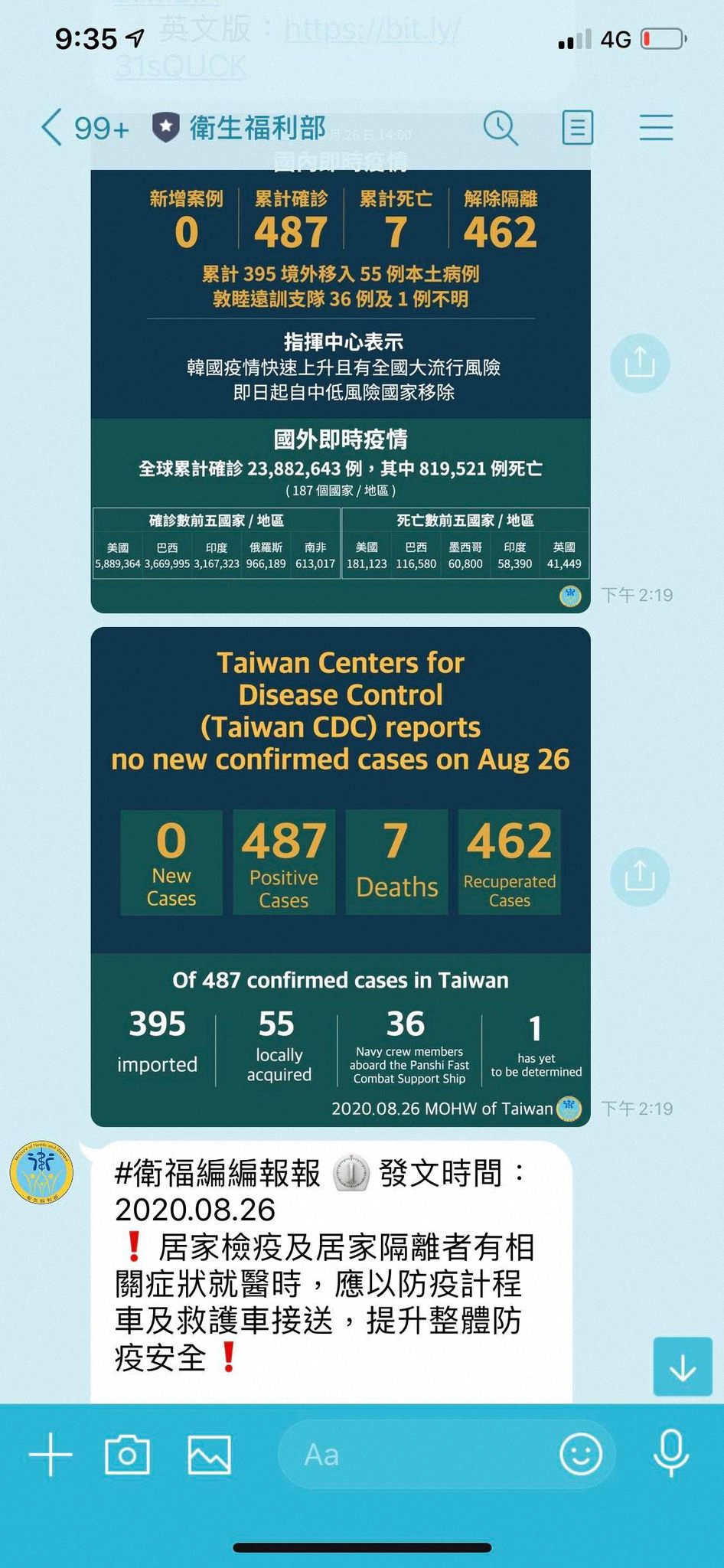

Supplement: Supplementary file 1 [file Image_1.jpg]

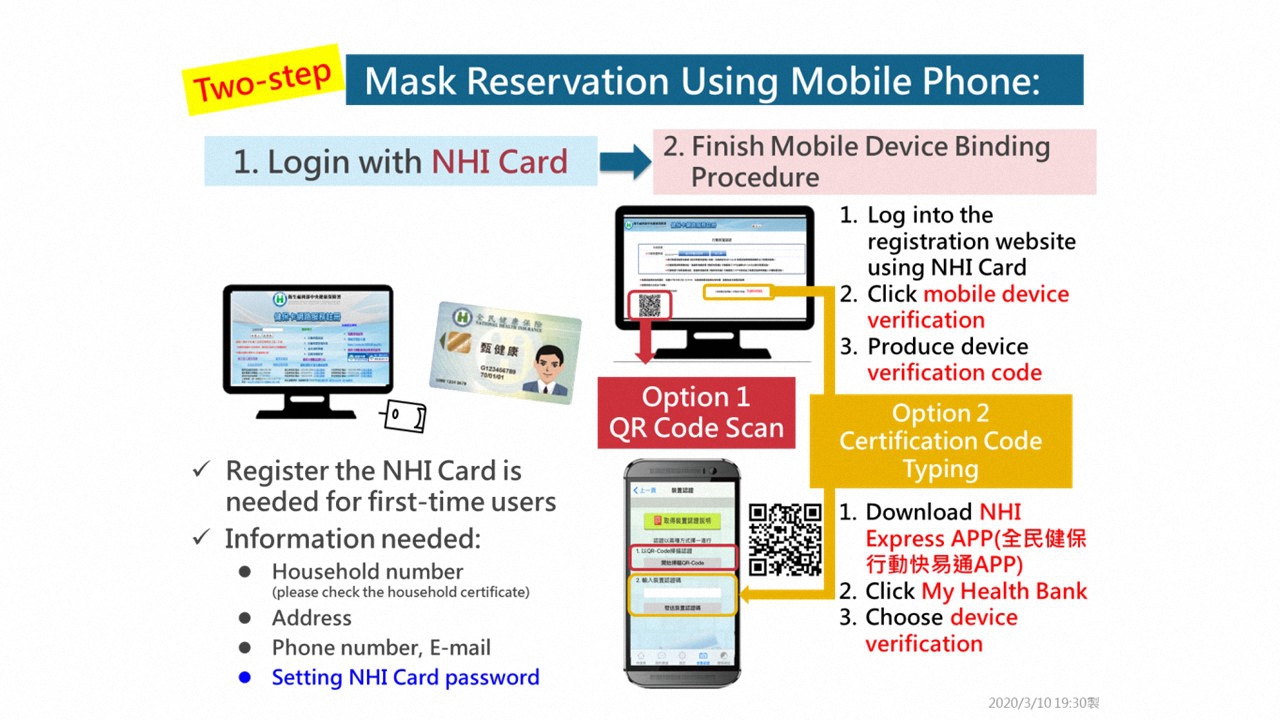

Supplement: Supplementary file 2 [file Image_2.jpg]

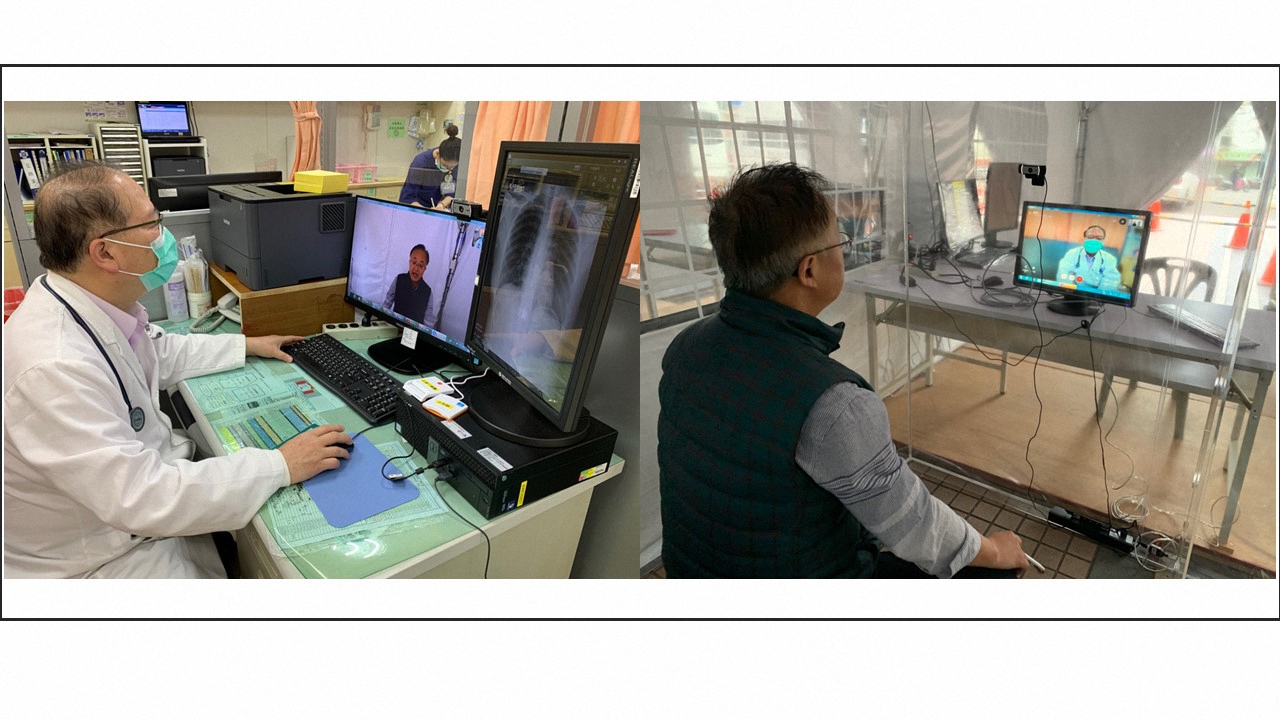

Supplement: Supplementary file 3 [file Image_3.jpg]

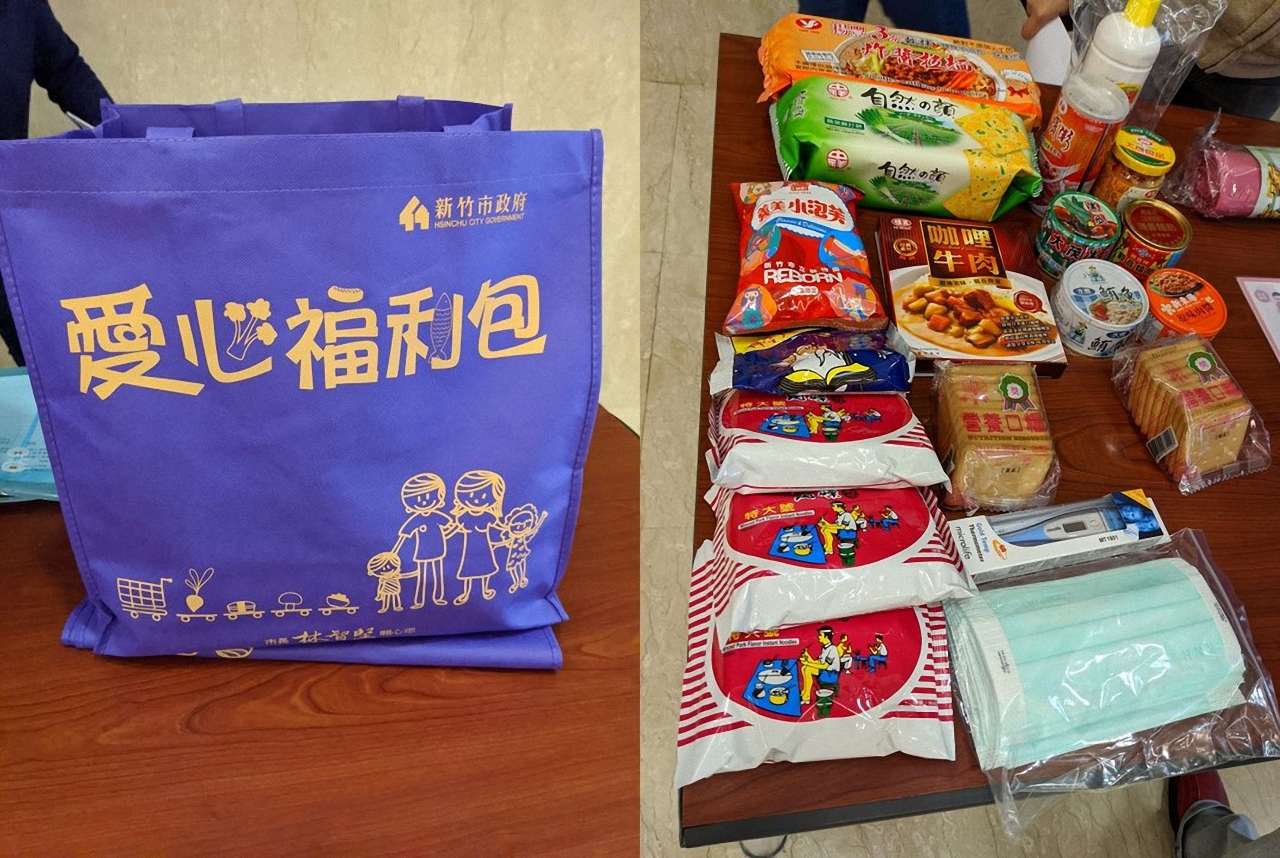

Supplement: Supplementary file 4 [file Image_4.jpg]

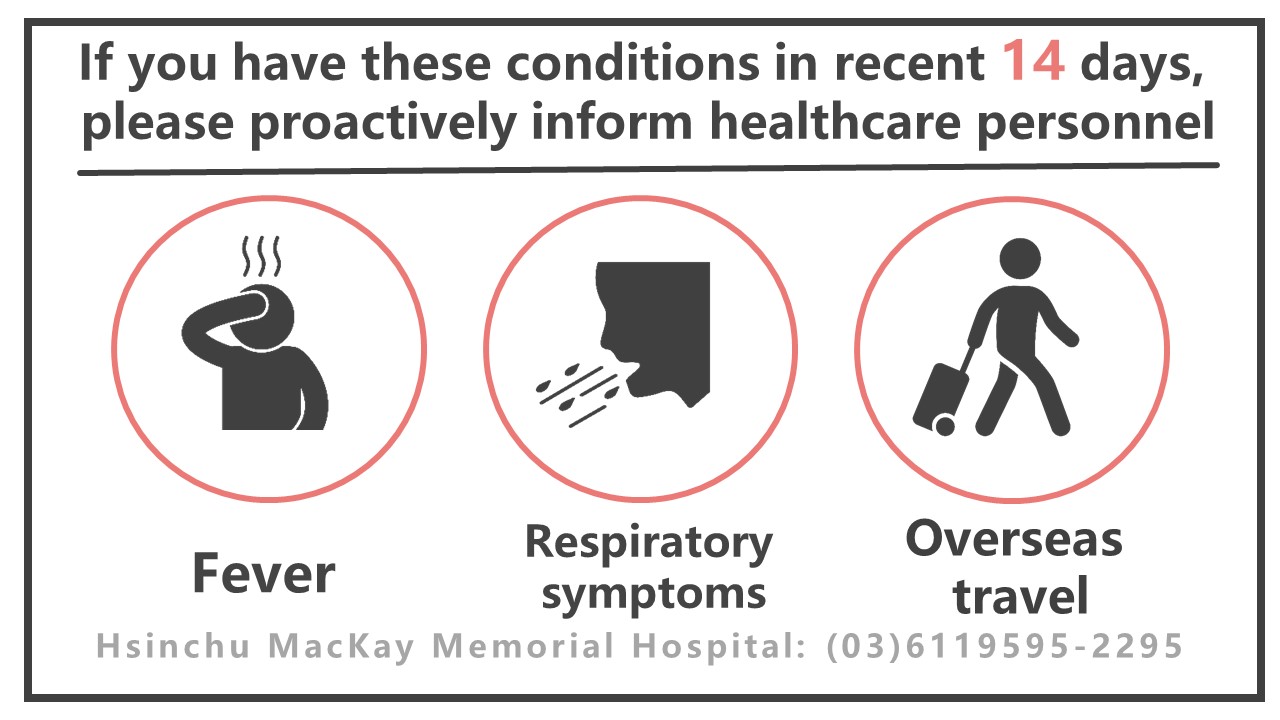

Supplement: Supplementary file 5 [file Image_5.jpeg]

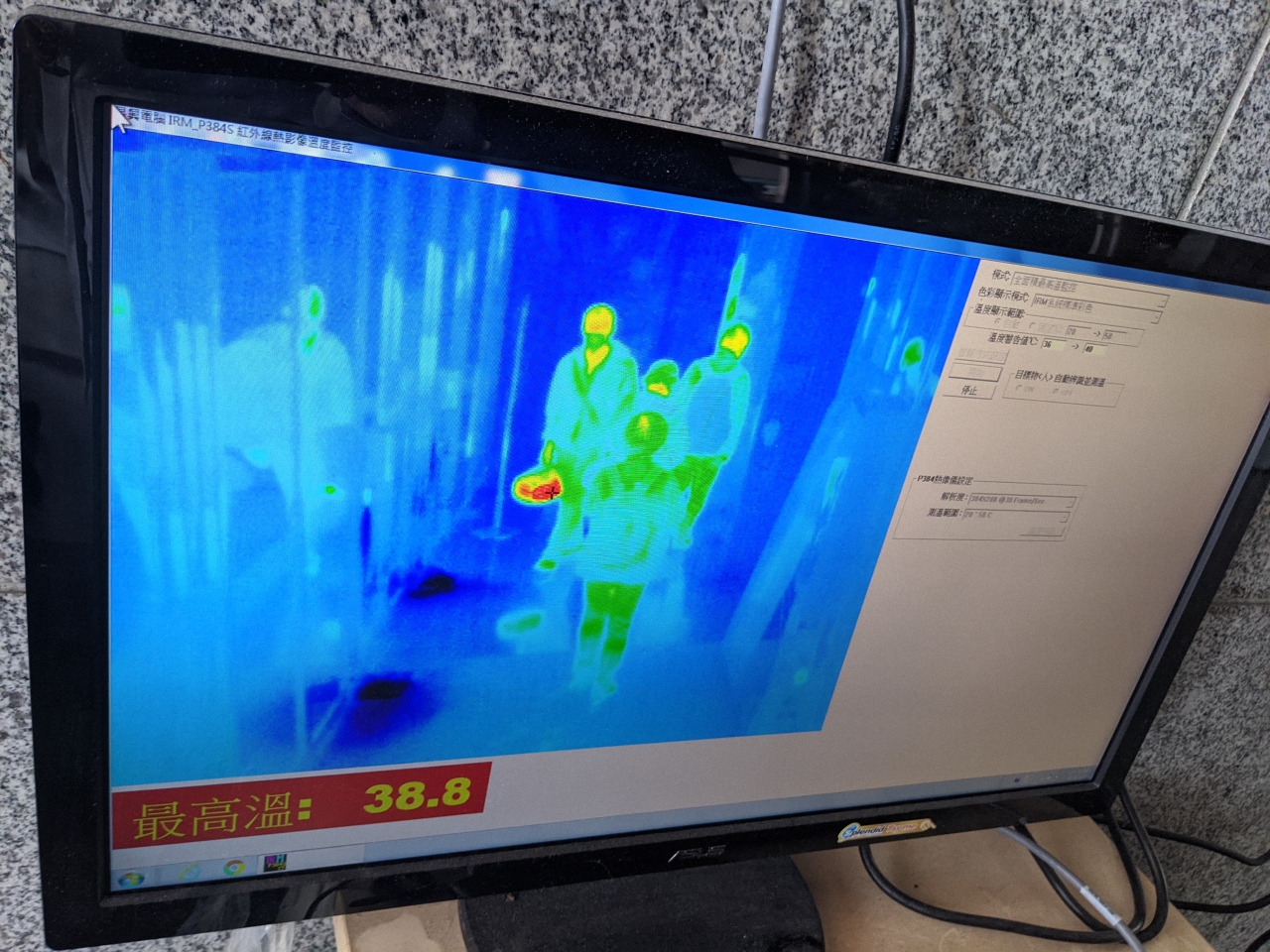

Supplement: Supplementary file 6 [file Image_6.jpg]
